# Supplementary material for: Post-systolic shortening index by echocardiography evaluation of dyssynchrony in the non-dilated and hypertrophied left ventricle
Source: PLoS One. 2022 Aug 25;17(8):e0273419. doi: 10.1371/journal.pone.0273419 (PMC9409501; doi:10.1371/journal.pone.0273419)
Supplement: S1 Table — (DOCX) [file pone.0273419.s001.docx]

**Supplemental Table 1.** Minimal dataset of this prospective cohort study.

| **Patient** | **Post systolic shortening index** | **Mechanical Dispersion index** | **Left ventricular global longitudinal strain (%)** | **QRS duration (ms)** |
| --- | --- | --- | --- | --- |
| 1 | 6.10 | 87.77 | -20.42 | 104 |
| 2 | 3.37 | 50.83 | -12.45 | 104 |
| 3 | 7.10 | 70.71 | -11.80 | 190 |
| 4 | 3.31 | 36.62 | -21.50 | 106 |
| 5 | 4.51 | 48.23 | -18.51 | 68 |
| 6 | 4.23 | 61.78 | -18.41 | 102 |
| 7 | 8.63 | 83.04 | -13.32 | 110 |
| 8 | 6.10 | 90.84 | -18.12 | 92 |
| 9 | 8.86 | 86.71 | -12.46 | 170 |
| 10 | 5.05 | 58.19 | -15.89 | 110 |
| 11 | 8.11 | 51.42 | -10.66 | 94 |
| 12 | 5.84 | 50.47 | -18.09 | 106 |
| 13 | 4.85 | 58.68 | -13.70 | 92 |
| 14 | 4.53 | 64.63 | -20.36 | 110 |
| 15 | 3.31 | 41.45 | -20.74 | 100 |
| 16 | 9.26 | 78.68 | -7.71 | 132 |
| 17 | 9.45 | 100.77 | -15.67 | 180 |
| 18 | 3.91 | 58.60 | -16.91 | 178 |
| 19 | 4.88 | 36.73 | -13.69 | 91 |
| 20 | 8.08 | 62.61 | -18.20 | 116 |
| 21 | 3.93 | 55.29 | -17.26 | 86 |
| 22 | 3.85 | 69.46 | -19.00 | 108 |
| 23 | 8.84 | 70.86 | -17.78 | 170 |
| 24 | 8.67 | 78.61 | -16.29 | 146 |
| 25 | 5.66 | 79.54 | -20.59 | 130 |
| 26 | 6.88 | 67.21 | -17.10 | 108 |
| 27 | 3.82 | 52.06 | -16.92 | 136 |
| 28 | 5.92 | 67.47 | -18.74 | 88 |
| 29 | 6.72 | 65.80 | -14.39 | 84 |
| 30 | 11.15 | 94.19 | -12.93 | 102 |
| 31 | 4.56 | 49.86 | -19.43 | 78 |
| 32 | 12.79 | 81.09 | -16.70 | 100 |
| 33 | 5.61 | 72.85 | -18.49 | 94 |
| 34 | 4.57 | 57.62 | -17.14 | 92 |
| 35 | 4.38 | 62.56 | -20.73 | 92 |
| 36 | 12.82 | 111.54 | -7.77 | 222 |
| 37 | 10.90 | 60.68 | -9.41 | 124 |
| 38 | 5.46 | 42.50 | -8.43 | 80 |
| 39 | 3.80 | 53.84 | -20.12 | 102 |
| 40 | 2.78 | 51.22 | -23.34 | 90 |
| 41 | 6.78 | 84.24 | -9.41 | 96 |
| 42 | 4.25 | 53.19 | -18.79 | 82 |
| 43 | 8.31 | 86.13 | -12.58 | 90 |
| 44 | 6.13 | 41.46 | -15.12 | 106 |
| 45 | 3.00 | 61.82 | -18.78 | 92 |
| 46 | 6.40 | 69.80 | -17.27 | 78 |
| 47 | 4.96 | 64.42 | -20.30 | 96 |
| 48 | 2.50 | 31.15 | -21.99 | 90 |
| 49 | 2.41 | 43.33 | -22.99 | 88 |
| 50 | 2.67 | 52.04 | -19.75 | 100 |
| 51 | 1.29 | 32.38 | -21.90 | 88 |
| 52 | 2.20 | 35.11 | -19.78 | 94 |
| 53 | 1.98 | 30.94 | -19.29 | 98 |
| 54 | 2.31 | 42.39 | -21.38 | 88 |
| 55 | 2.52 | 33.31 | -22.43 | 116 |
| 56 | 2.96 | 31.19 | -22.64 | 88 |
| 57 | 1.40 | 37.03 | -21.31 | 88 |
| 58 | 1.97 | 45.83 | -19.13 | 84 |
| 59 | 2.12 | 48.61 | -21.75 | 100 |
| 60 | 1.07 | 32.91 | -23.53 | 94 |
| 61 | 2.99 | 47.29 | -20.36 | 84 |
| 62 | 1.24 | 30.66 | -21.58 | 80 |
| 63 | 2.89 | 32.92 | -18.20 | 90 |
| 64 | 4.41 | 58.27 | -19.73 | 134 |
| 65 | 5.72 | 51.46 | -21.58 | 70 |
| 66 | 7.15 | 77.04 | -9.71 | 94 |
| 67 | 5.51 | 56.78 | -14.29 | 82 |
| 68 | 6.63 | 35.41 | -11.30 | 72 |
| 69 | 6.95 | 61.82 | -11.30 | 188 |
| 70 | 13.51 | 73.09 | -12.99 | 110 |
