# Supplementary material for: Post-systolic shortening index by echocardiography evaluation of dyssynchrony in the non-dilated and hypertrophied left ventricle
Source: PLoS One. 2022 Aug 25;17(8):e0273419. doi: 10.1371/journal.pone.0273419 (PMC9409501; doi:10.1371/journal.pone.0273419)
Supplement: Attachment — Submitted filename: Responses to reviewer (3).docx [file pone.0273419.s002.docx]

**Responses to reviewer/editor comments**

**ACADEMIC EDITOR:**

The authors should more focus on the clinical implications and perspectives. Moreover, they should discuss the role of regional left ventricular dyssynchrony related to potential myocardial hibernation in the light of previous unmentioned pre-clinical study (please see J Card Fail. 2009 Dec;15(10):920-8).

Response: We have added the following to the discussion: “Usually, electromechanical dyssynchrony and regional LV dysfunction brought by perfusion abnormality are thought of as separate mechanisms with different “phenotypes”. Yet, the two processes of dyssynchrony and hypoperfusion can coexist and reinforce each other. Lionetti et al showed that, in an animal model, LV dyssynchrony induced by pacing leads to hibernation [ref]. Prior studies have also identified PSI as a potential imaging biomarker for ischemia. We provide further proof of intrinsic link between temporal parameters of regional dyssynchrony such as MDI and parameters of amplitude of segmental wall contraction such as PSI.

**Reviewer #1:**

This is the first study to report about the presence of PSI in patients with HCM and amyloidosis, showing that mean PSI value of all software products in the setting of these population significantly increased compared with controls. Congratulation to the authors, here you find some comments in order to improve the manuscript
Response: We thank reviewer 1 for the positive and insightful review to improve our manuscript.

Discussion: It is clear how patients with HCM may have more pathologic post-systolic shortening, which may have etiologic contribution to the functional heterogeneity of this disease entity especially diastolic dysfunction (DOI: 10.1016/j.echo.2006.03.019). In this scenario authors should also focus more on the considered HCM and amyloidosis diagnostic criteria. Please add a nice figure

Response: This is a good point regarding the heterogeneity between HCM and cardiac amyloidosis from the morphological and functional perspective. Unfortunately with our prospective cohort of only 36 HCM and 18 amyloidosis patients the sample size had insufficient power to evaluate the discriminative value of PSI to identify HCM versus amyloidosis. Analogous to strain, global LV measures like GLS and PSI may not be able to distinguish the two as much as segmental and relative ratios of regional strain and PSI, which is beyond the scope of this study. The mentioned article has already been referenced. We have provided an additional part to Figure 3 (see below) to show examples of the average segmental PSI plot for controls, HCM (non-apical and apical) and cardiac amyloidosis.

In discussion please explain author’s thoughts about post-systolic shortening in “grey zones population” such subjects with athlete’s heart but no clear diagnosis of cardiomyopathy (please cite: DOI: 10.1111/jce.14526). In fact, today distinguishing hypertrophic cardiomyopathy from athlete’s heart represents a clinical problem of increasing magnitude and significance (doi: 10.1136/hrt.2005.060962; DOI: 10.1016/j.ijcard.2021.10.013). Is PSS minimally influenced by dyssynchrony that is intrinsic to the presence of physiologic LV hypertrophy? Please discuss and cite 4 suggested references

Response: We have added the following to the discussion: “Another area of potential use of PSS is assessment in “grey zone populations” such as LV hypertrophy in athletes with no clear diagnosis of cardiomyopathy (DOI: 10.1111/jce.14526). Distinguishing hypertrophic cardiomyopathy from athlete’s heart represents a clinical problem of increasing magnitude and significance (doi: 10.1136/hrt.2005.060962; DOI: 10.1016/j.ijcard.2021.10.013). While no data are available on the impact of athlete’s heart on PSS (or myocardial dispersion), one can speculate that these patients have preserved long axis function, and thus less so PSS. Further studies are needed to assess this issue.”

It is definitely not clear how many patients underwent to CMR (providing best comprehensive characterization of the HCM, cite DOI: 10.1016/j.jcmg.2019.09.020) to genetic testing (DOI: 10.3390/ijms221910401) . Moreover how many patients were symptomatic? (the importance of syncopal diagnosis due to LVOT obstruction), did you find a difference in your patient population? Please explain in the discussion/limitations the importance of these topics, why you did not investigate and cite all suggested references

Response: Thank you very much for your suggestion. The aim of our study was not to differentiate between various types of HCM, or between gene-positive and gene-negative phenotypes, but to assess the impact of HCM on PSI across varying phenotypes. In that sense, using PSI to differentiate between presence or absence of dynamic LVOT obstruction, or symptoms of any kind, is less relevant then establishing that HCM is associated with PSI, and that the magnitude of that association depends on the software used to assess PSI. In addition, our study sample was too small to accurately differentiate between phenotypes. Nevertheless we have added the following as suggested into the methods section first paragraph: “All HCM and amyloidosis patients were symptomatic. Amongst HCM patients, diagnosis was confirmed by cardiac magnetic resonance in 29(DOI: 10.1016/j.jcmg.2019.09.020), and by subsequent operative histology findings in 3 patients, while the remaining were diagnosed by echocardiography alone (3 because of having an implantable cardiac defibrillator). Eleven patient underwent genetic testing, with pathogenic mutation present in 7 subjects (DOI: 10.3390/ijms221910401).” Cardiac amyloidosis diagnosis was defined in our previous study in the methods section (ref): *Patients in the cardiac amyloidosis cohort carried this diagnosis based upon endomyocardial biopsy or a non-invasive diagnosis centered around cardiac imaging. Patients without endomyocardial confirmation of immunoglobulin light chain (AL) amyloidosis had an extra-cardiac biopsy consistent with the disease as well as typical echocardiographic and/or cardiac magnetic resonance imaging features. Patients without endomyocardial biopsy confirmation of transthyretin (ATTR) amyloidosis had technetium pyrophosphate nuclear scintigraphy and/or cardiac magnetic resonance imaging features consistent with the disease. Patients in the HCM cohort were diagnosed based upon history, examination, electrocardiography, and other multimodality imaging using the latest societal guidelines to diagnose HCM. Septal and apical variants of HCM define the region of maximal hypertrophy which have been shown to display differing regional variations in LS. Patients with apical aneurysms were excluded. A control group of patients was recruited without valvular, structural, or coronary artery disease and normal systolic and diastolic function.*

In the paragraph “Clinical implication” more author’s considerations among MDI and PSI as potentially useful for assessing the risk stratification of ventricular arrhythmias, are welcome. Moreover how would you follow these patients? Holter? Loop recorder? Only symptoms? Please explain

Response: As discussed in clinical implications with now updated edits: “PSI and MDI in patients with HCM and amyloidosis are potentially useful for assessing the risk stratification of ventricular arrhythmias, as it seems that shortening after aortic valve closure reflects segmental myocardium dysfunction or electrical conduction dysfunction. Further studies are warranted to evaluate the association of increased PSI with ventricular arrhythmia and sudden death events in these populations. If these associations exist, PSI and/or MDI should be routinely assessed in these patients when echocardiography is performed, and if abnormally elevated then more frequent heart rhythm monitoring may be considered”
